# The Roles of the Wnt-Antagonists Axin and Lrp4 during Embryogenesis of the Red Flour Beetle *Tribolium castaneum*

**DOI:** 10.3390/jdb5040010

**Published:** 2017-10-15

**Authors:** Romy Prühs, Anke Beermann, Reinhard Schröder

**Affiliations:** 1 Department of Genetics, Biological Sciences, University of Rostock, Albert-Einstein-Str. 3, D 18059 Rostock, Germany; romy.pruehs@gmx.de; 2 Animal Genetics, University of Tübingen, Auf der Morgenstelle 15, D 72076 Tübingen, Germany; anke.beermann@t-online.de

**Keywords:** *Tribolium*, Wnt signaling, Wnt-antagonist, Axin, LRP4, AP-polarity, double abdomen

## Abstract

In both vertebrates and invertebrates, the Wnt-signaling pathway is essential for numerous processes in embryogenesis and during adult life. Wnt activity is fine-tuned at various levels by the interplay of a number of Wnt-agonists (Wnt ligands, Frizzled-receptors, Lrp5/6 coreceptors) and Wnt-antagonists (among them Axin, Secreted frizzled and Lrp4) to define anterior–posterior polarity of the early embryo and specify cell fate in organogenesis. So far, the functional analysis of Wnt-pathway components in insects has concentrated on the roles of Wnt-agonists and on the Wnt-antagonist Axin. We depict here additional features of the Wnt-antagonist Axin in the flour beetle *Tribolium castaneum*. We show that *Tc-axin* is dynamically expressed throughout embryogenesis and confirm its essential role in head development. In addition, we describe an as yet undetected, more extreme *Tc-axin* RNAi-phenotype, the ectopic formation of posterior abdominal segments in reverse polarity and a second hindgut at the anterior. For the first time, we describe here that an *lrp4* ortholog is involved in axis formation in an insect. The *Tribolium* Lrp4 ortholog is ubiquitously expressed throughout embryogenesis. Its downregulation via maternal RNAi results in the reduction of head structures but not in axis polarity reversal. Furthermore, segmentation is impaired and larvae develop with a severe gap-phenotype. We conclude that, as in vertebrates, *Tc-lrp4* functions as a Wnt-inhibitor in *Tribolium* during various stages of embryogenesis. We discuss the role of both components as negative modulators of Wnt signaling in respect to axis formation and segmentation in *Tribolium*.

## 1. Introduction

During embryogenesis and adult life, the precise regulation of the Wnt-signaling pathway is essential for a wide variety of processes like cell-fate specification, axis formation, tissue and organ development. In the canonical or cell-fate pathway, signaling is triggered when a Wnt-ligand binds to a Frizzled (Fz) receptor and a coreceptor, the low-density lipoprotein receptor-related protein (Lrp) 5, 6 or Arrow. This ternary complex represents the “ON”-status and leads to the disintegration of the β-catenin destruction complex by recruiting one of its components, Axin, to the cell membrane. As a consequence, β-catenin becomes stabilised in the cytoplasm and subsequently undergoes nuclear localization to regulate Wnt-target genes. In the absence of a Wnt-ligand (“OFF”-status), β-catenin has a very short half-life in the cytoplasm as it becomes marked for destruction by the proteasome by components of the destruction complex APC, Axin, Gsk3β and CK1α [1,2]. The active Wnt pathway regulates the increase in cell proliferation and motility [3], and unregulated elevated Wnt activity can eventually result in various diseases, including the formation of tumours. Thus, the negative modulation of Wnt activity is equally important for tissue differentiation and homeostasis. In vertebrates, an array of Wnt-inhibitor molecules is known that antagonistically interfere with this signaling pathway at various levels, like Wnt-secretion, intracellular Wnt-transport, Wnt-modification and Wnt-reception. At the receptor level, Wnt-induced Fz-Lrp 5/6 dimerization is antagonized by proteins such as WISE, SOST or DKK [4]. Another way to downregulate Wnt activity is sequestering Wnts by Wnt-binding proteins that are unable to transmit the signal to the nucleus and, in this way, limiting the number of Wnt-ligands available for the Fz-receptor. To this class of “dominant-negative” acting receptors belong the secreted frizzled-related proteins (sFRPs) and Lrp4 [5]. Decreasing the function of Wnt-inhibitors either experimentally or by mutation results in the upregulation of Wnt activity, a situation seen in various forms of cancer [4]. One important step in early embryogenesis it the establishment of anterior–posterior polarity of the body axis and of the nervous system [2,6,7]. In both normal development and during regeneration, high levels of Wnt activity are required for the specification of posterior fate, while the absence of Wnt signaling enables the development of anterior structures in all extant animals [8,9,10,11]. At the posterior, a Wnt-signaling centre is established through expressed Wnt-ligands that activates targets like Brachyury (Byn) expression, which itself activates Wnt signaling. In this way, a self-perpetuating Wnt–Byn circuit is established that maintains posterior fate [2]. Consequently, activating Wnt signaling ectopically at the anterior pole results in the development of ectopic tail structures at the expense of anterior ones. To allow for the specification of anterior fate, Wnt activity has to be efficiently downregulated in regions where head—or more generally—anterior development should take place. This can be ensured by anteriorly expressed Wnt-antagonists. Most of such antagonists known from vertebrates are, however, absent from the genome of insects. In the short germband insect *Tribolium*, Wnt-ligands are expressed at the posterior of the early blastoderm embryo [12] and required for axial elongation [13,14,15]. Breaking the symmetry of Wnt activity along the main body axis in *Tribolium* is achieved by the anterior asymmetric expression of Axin, a crucial component of the destruction complex. Here *Tc-axin* is required for keeping the anterior egg free of Wnt activity and in this way, allows for head development. Consequently, downregulation of *Tc-axin* resulted in severely affected embryos consisting of posterior abdominal segments only [16]. The *Drosophila* Lrp4 ortholog is expressed in the adult brain and functions independently of a Wnt-ligand in synaptogenesis [17]. Lrp4 in vertebrates can interact with Wnts [18] and other ligands and has been characterized as a Wnt-modulator in various processes such as the development of skin-appendages [19], as well as bone [20] and tooth formation [21]. 

Here, we extend a previous study on the Wnt-antagonist *axin* in *Tribolium* [16] and show that, also later during embryogenesis, distinctly expressed *Tc-axin* defines regions where Wnt activity is modulated. Furthermore, we show here that the downregulation of *Tc-axin* results in ectopic formation of a second hindgut and the development of an ectopic tail with reversed polarity at the anterior pole, resembling the *bicoid* phenotype in *Drosophila* [22]. We also have characterised another antagonist of Wnt activity, the *Tribolium* Lrp4-ortholog. In contrast to *Tc-axin*, *Tc-lrp4* is ubiquitously expressed in the egg and throughout later embryogenesis. RNAi knockdown of *Tc-lrp4* resulted in anterior truncations, as observed with *Tc-axin* RNAi. Interestingly, strong segmentation defects seen are likely caused by the upregulation of segment polarity genes. 

Together, we demonstrate here, that the concerted interaction of Wnt-agonists, and Wnt-antagonists fine-tune early pattern formation in a short germband insect.

## 2. Materials and Methods

**Animal husbandry**. Beetles were reared on a regular changing diet of whole wheat and instant flour in a 30 °C incubator.

**Molecular biology**. DNA fragments corresponding to the respective genes were amplified from cDNA by PCR, and cloned according to standard procedures. Each clone was verified by sequencing (LGC-Genomics). Injections of dsRNA generated from non-overlapping constructs were performed to avoid off-target effects. All tested dsRNA showed the same range of phenotypes for the respective gene. Injections were usually performed into the wild-type strain (San Bernadino) as described [23].

dsRNA generation used for parental RNAi. The position of the fragments relative to the exon–intron structure of *Tc-axin* is indicated in Figure S1A. dsRNA for *Tc-axin* (TC006314): *axin*-iB1 (position coding sequence 184–695) and *axin*-iB2 (position cds 990–1213) were obtained from a company (Eupheria) [16]. One *Tc-axin* cDNA-clone (position cds 523–1344) partially overlapping with both *Tc-axin*-iB fragments served as a template for the generation of dsRNA. We injected various concentrations of *Tc-axin*-dsRNA (10–1000 ng/μL) and got the full range of phenotypes between 20 ng/μL and 50 ng/μL. Results of *axin* RNAi were also reproduced in another beetle strain EFA-nGFP [24] to exclude strain-specific background effects.

The *Tc-lrp4* ortholog (TC007146) is present on the minus-strand on ChLG4. The position of cloned cDNA fragments relative to the gene structure is indicated in Figure S2A. The following primers were used (position number according to the coding sequence): *Tc-lrp4* #1: fw: GGACGCGCTACCCCTTTGA (473–491); rev: CGCCGTCGCATTTGAACTTTA (1223–1243), fragment length 771bp; *Tc-lrp4* #2: fw: ACCGCGCTGATAAAACCAAT (4448–4467); rev: TCGCGTCAATCCAATAGAGG (5205–5224)), fragment length 777bp; *Tc-lrp4* #3 (iBeetle fragment iB_01131): fw: GTGTAGAAGGCGAACCCAAA (5564–5583); rev: CAGTTGGAGCTGCAAGGAGT 6019–6038), fragment length 475bp. Concentrations of *Tc-lrp4*-dsRNA varied from 340 ng/μL to 3 μg/μL. Details are listed in Supplementary Materials (Tables S1–S3). The same low amount of wild-type cuticles and all phenotypic classes have been observed with all concentrations of dsRNA tested. Eggs and embryos used for marker-gene analyses came from females injected with dsRNA with a concentration of 800 ng/μL.

Phenotypic analysis. The larval cuticle phenotype was inspected making use of their autofluorescence under UV light to visualize all cuticularised structures including the gut. In situ hybridizations and antibody staining on wild-type and RNAi embryos were performed as described previously [25]. Each probe/antibody was carefully titrated to ensure an optimal signal-to-background ratio.

Analysis of protein-structure was performed using SMART (http://smart.embl-heidelberg.de) or motif-search (www.genome.jp/tools/motif/).

## 3. Results

### 3.1. The Wnt-Inhibitor Tc-axin Is Asymmetrically Expressed in the Egg and Dynamically with Distinct Patterns during Further Embryogenesis

**Figure 1:**
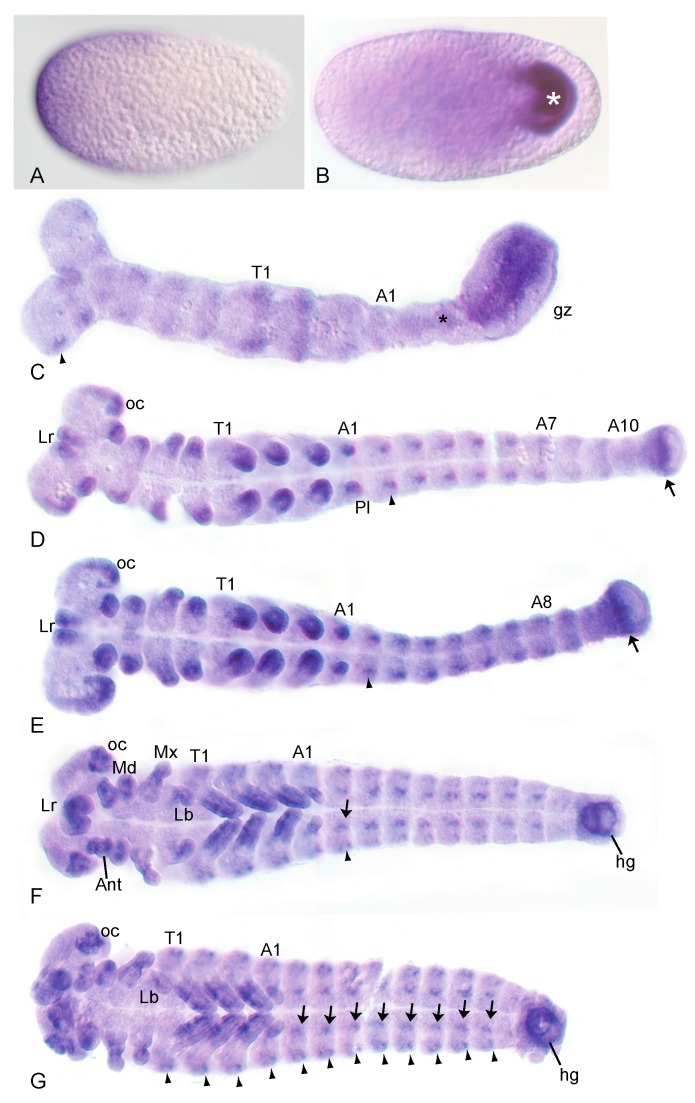
*Tc-axin* shows a distinct, dynamic expression pattern throughout embryonic development. *Tc-axin*-mRNA localisation by in-situ hybridisation in wild-type embryos. (**A**) Early cleavage stage embryo with asymmetric anterior expression of *Tc-axin* mRNA (anterior points to the right). (**B**) Gastrulating embryo (ventral view) with strong upregulation of *Tc-axin* expression in the posterior invaginating cells (star). (**C**) Embryo undergoing germband extension. A longitudinal *Tc-axin* domain within the growth zone shows slight upregulation at its border. Weak expression is seen along the midline within the presegmented region (star). In addition, segmental stripes within thoracic segment 1 and 2 and spotted domains (arrowhead) within the head are visible. (**D**,**E**) Fully segmented embryos with expression of *Tc-axin* in the distal parts of the head-, gnathal- and thoracic appendages and in the pleuropodia, the appendages of abdominal segment 1. Within each abdominal segment a pair of *axin*-expressing cells become apparent (arrowhead). Arrow marks cells likely belonging to the hindgut anlage. (**F**,**G**) In the retracting germband, *Tc-axin* is expressed ventrally in the thoracic legs while in the other appendages no regionalized expression is seen. Expression strength diminished in the maxillae (Mx) and the labral appendages (Lr). During formation of the hindgut (hg), *Tc-axin* was seen around the future proctodeal opening. Within each abdominal segment as well as in the thorax, a further *axin* expression domain marks a group of lateral cells (arrowhead) in addition to *axin*-positive cells near the midline (arrow). In the fully segmented embryo, the ocular region of the head (oc) permanently expresses *Tc-axin* (**D**–**G**). A: abdominal segment; gz: growth-zone; hg: hindgut; Lb: labium; Lr: labrum Md: mandible; Mx: maxilla; oc: ocular segment; Pl: pleuropodium.

As described previously [16], the initial expression of the Wnt-inhibitor *Tc-axin* (*Tc-ax*) was observed asymmetrically in a small anterior crescent of a freshly laid egg (Figure 1A), and more broadly covers the anterior third of the egg throughout the early cleavage stages. At the beginning of gastrulation, *Tc-axin* was lost from the anterior region and expression was seen in the posterior growth zone of the young germ rudiment (Figure 1B). During axis elongation, *Tc-axin* remained strongly expressed in a longitudinal domain within the growth zone and weakly along the midline in the non-segmented region anterior to the growth zone, the presegmented region (Figure 1C). Within the thorax and the head, weak additional *Tc-axin* expression domains in the limb fields were visible. During segmentation of the germband, *Tc-axin* expression sites became stronger and more pronounced in the head lobes and in the distal parts of the outgrowing appendages of the gnathum and the thorax, while in the abdominal segments new dot-shaped expression domains appeared. At this stage, *Tc-axin* expression at the posterior is confined to a subterminal stripe (Figure 1D,E). After germband retraction, *Tc-axin* was seen ventrally in the thoracic appendages as well as in the anlagen of the hindgut (Figure 1F,G). The expression domains within the distal head lobes and the gnathal appendages remained while in each segment one inner and one outer pair of *Tc-axin* positive groups of cells were visible (Figure 1G).

### 3.2. Reduction of Tc-axin Function Results in Anterior Truncated, Mild Bicaudal and Symmetric Double Abdomen Embryos

**Figure 2:**
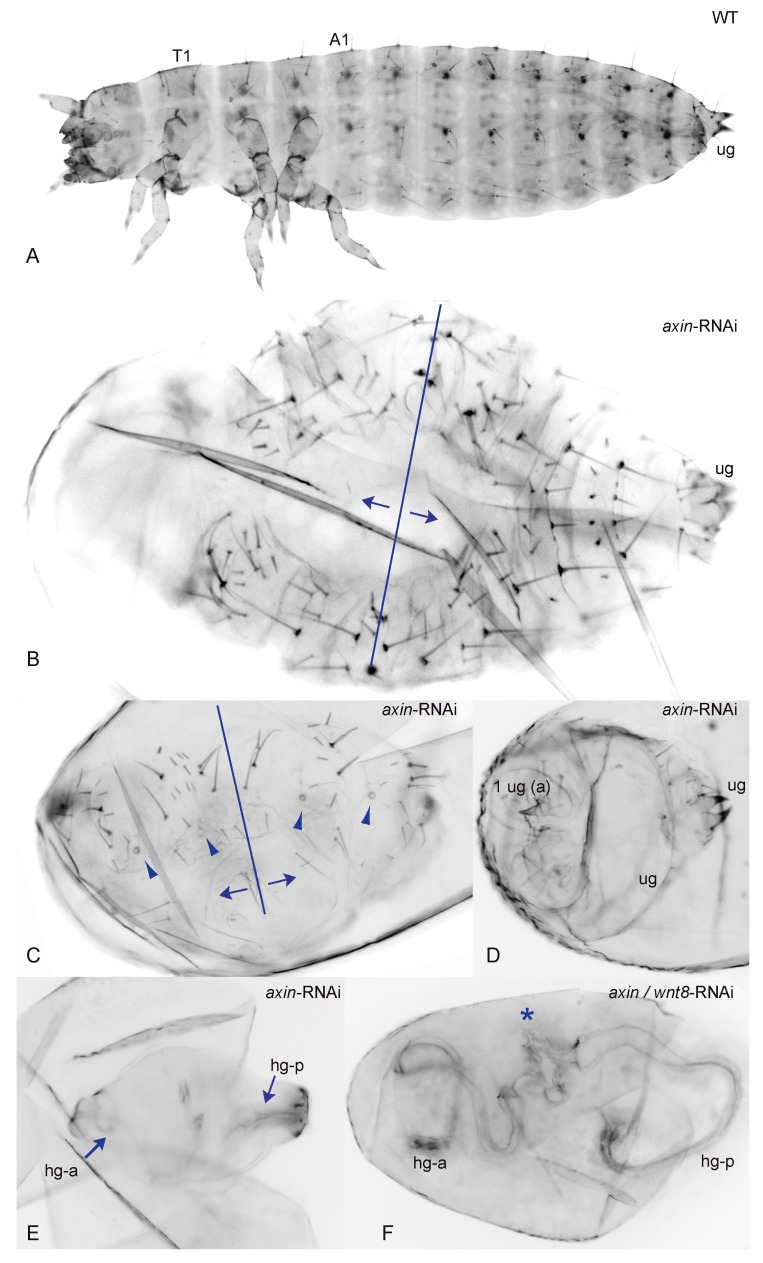
Polarity reversal in *Tc-axin*^RNAi^ and *Tc-axin*/*wnt8*^RNAi^ cuticles. (**A**) Wild-type cuticle (ug: urogomphi) (**B**–**E**) *Tc-axin*^RNAi^ polarity reversal phenotype. (**B**,**C**) Arrows indicate the polarity of the segments as judged by the orientation of the larger bristles that always point to the posterior pole. Plane of polarity reversal is indicated (line). (**B**) Posterior abdomen consists of five segments; at the anterior, at least three abdominal segments of reversed polarity can be identified. (**C**) Mirror-image double abdomen with two abdominal segments at each half. Arrowheads point to the tracheal openings. (**D**) One additional urogomphus (ug) has formed at the anterior. (**E**) Symmetric *axin*-RNAi cuticle of the double-abdomen phenotype with two hindguts on opposing sites of the unsegmented cuticle. (**F**) *axin*-*wnt8* double-RNAi: cuticle remnant (star) with two hindguts (hg-a, hg-p).

By knocking down *Tc-axin* through RNA interference, we obtained embryos and larval cuticles missing anterior pattern elements and classified them according to their phenotypic strength in two different classes. When compared to the wild-type cuticle (Figure 2A), class I *Tc-axin*^RNAi^ embryos and larvae were identified by the loss of anterior structures to various degrees ranging from reduced head parts only, the loss of the head and some thoracic segments to the loss of all anterior segments including head, thorax and the anterior part of the abdomen (Figure S3); see also [16].

Embryos and cuticles belonging to the second class showed a different phenotype: instead of anterior structures, posterior abdominal segments with opposing polarity formed anterior to abdominal segments with original orientation (Figure 2B). Such polarity reversal along the AP-axis was classified as a mild double-abdomen (DA) phenotype. A weak, asymmetric DA phenotype is marked by the polarity-reversal of abdominal bristles belonging to one or two abdominal segments at the anterior of an otherwise normally formed abdomen with fewer or the normal number of segments (Figure 2B). A more distinct phenotype was represented by cuticles with complete mirror symmetry that displayed two partial posterior abdomens opposing each other (Figure 2C–F). In such cuticles, each half consisted of 2–3 abdominal segments where the ectopically formed, duplicated abdomen mostly also included internal structures like the hindgut (Figure 2E,F). The most extreme double abdomen phenotype displayed only remnants of a posterior cuticle, including two hindgut tubes that formed at opposite sites, and was seen not only in *Tc-axin*^RNAi^ but also in *axin*-*Wnt8* double RNAi experiments (Figure 2F). 

Since 46% N = 185) of the analysed eggs were empty (Table 1) and did not develop to an analysable cuticle, we asked whether the formation of the ectopically formed abdomen was the result of early patterning events or is caused only secondarily later in development during germband elongation or cuticle formation. Therefore, we analysed the expression of posteriorly expressed genes as well as segmental marker genes in *Tc-axin*^RNAi^ embryos. First, we visualized the read-out of the Wnt-signaling pathway in *Tc-axin*^RNAi^ embryos by looking for the expression of *Tc-caudal*, *Tc-brachyenteron*, *Tc-wnt8* and *Tc-AbdB* as marker genes.

**Figure 3:**
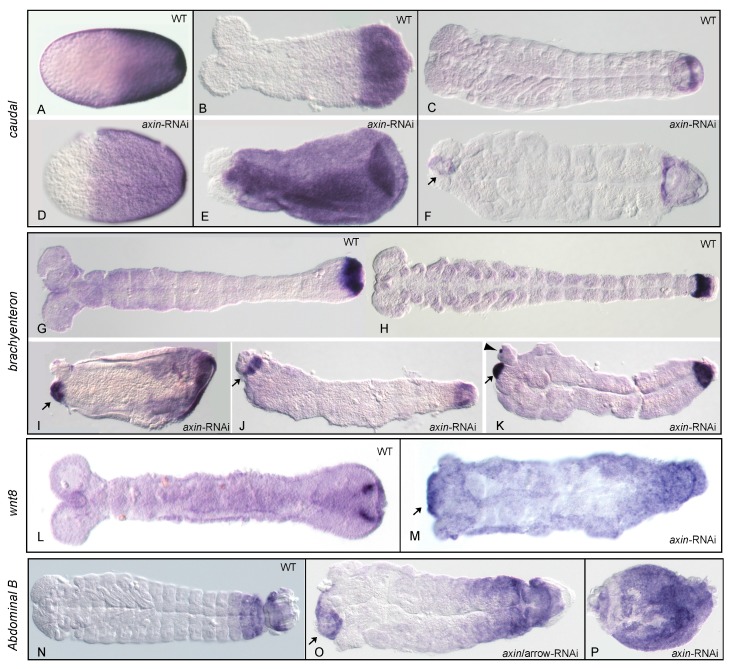
Analysis of posterior marker gene expression in *Tc-axin*^RNAi^ embryos. (**A**–**C**) *Tc-caudal* expression in successively older wild-type embryos (**D**) *Tc-axin*^RNAi^ blastoderm embryo: anterior expansion of *Tc-caudal* expression covering the embryonic region. (**E**) *Tc-caudal* is highly overexpressed in a *Tc-axin*^RNAi^ embryo leaving free only the most anterior tissue. (**F**) *Tc-axin*^RNAi^ embryo after completion of segmentation: ectopic *Tc-cad* expression at the anterior (arrow). (**G**,**H**) In the wild-type, *Tc-brachyenteron* (*Tc-byn*) is exclusively expressed in the precursor of the hindgut at the posterior. (**I**–**K**) *Tc-axin*^RNAi^ embryos showing ectopic *Tc-byn* expression at the anterior (arrow). (**I**) *Tc-byn* expression is strongly expanded at the posterior of a young germ anlage. (**K**) Two ectopic *Tc-byn* domains are visible at the anterior (arrow, arrow-head); (**L**) *Tc-wnt8* is detectable in two spots in the growth zone in the wild-type but diffuse throughout a *Tc-axin* RNAi-embryo (**M**) and upregulated at the anterior (arrow). (**N**) *Tc-Abdominal B* is expressed in the posterior segments and the hindgut anlagen in the wild-type. (**O**) *Tc*-*axin-arrow*-double RNAi: *AbdB* expression is seen expanded in the posterior abdomen and ectopic at the anterior (arrow). (**P**) *Tc-axin*-RNAi upregulated throughout the embryo.

Posteriorization of early *Tc-axin*^RNAi^ embryos was documented by the expansion of the Wnt-target gene *Tc-caudal* already in the blastoderm embryo (compare Figure 3A,D). In contrast to the wild-type, where *Tc-caudal* is expressed in the posterior half between 44% and 58% egg-length (Figure 3A, mean: 51.5%, posterior pole = 100% egg length; N = 23) [26], *Tc-caudal* expression in *Tc-axin*^RNAi^ blastoderm embryos was expanded to the anterior to 63% egg-length (Figure 3D, range: 57–73%; N = 24) and covered nearly the complete prospective embryonic region of the blastoderm embryo. However, in wild-type embryos of the germband stage, *Tc-caudal* expression was restricted to the posterior growth zone (Figure 3B), but was distributed within nearly the complete embryo after *Tc-axin-*RNAi (Figure 3E). Later, in the fully segmented wild-type embryo, *Tc-caudal* expression was restricted to a small band in the posterior gut region (Figure 3C), but, after *Tc-axin-*RNAi, was also detected ectopically at the anterior indicating the formation of tissue with hindgut properties (Figure 3F). Indeed, the exclusive marker for the hindgut precursor *Tc-brachyenteron* (Figure 3G,H) [27] was also observed ectopically at the anterior in *Tc-axin*^RNAi^ embryos, either in a single domain (N = 8/20) or in two spots (12/20) at the newly formed posterior end (Figure 3I–L), otherwise never observed in wild-type embryo. In the *Tc-axin*^RNAi^ embryo of the early germband stage, *Tc-byn* expression itself was highly upregulated and encompassed the complete posterior region (Figure 3I). In the single *Tc-axin*-RNAi experiment, the strongest double abdomen phenotype (two opposing hindguts, Figure 2F) in the cuticle preparations showing polarity reversal was obtained with the frequency of 47% (N = 9, Figure S4). That Wnt activity, indeed, was elevated in *Tc-axin*-RNAi embryos and was visualised by the ubiquitous expression in the germband and with the ectopic upregulation at the anterior of the embryo (Figure 3M). By analysing *Tc-Abdominal B,* we could also show that posterior expression domain was slightly expanded and an ectopic domain formed at the anterior in weaker affected embryos (Figure 3O). Embryos with a stronger RNAi-phenotype showed nearly ubiquitous *Tc-AbdB* expression (Figure 3P). We then asked whether the frequency of the strongest double abdomen phenotype could be enhanced, and carried out *Tc-axin* RNAi in combination with knocking down *Tc-wnt8* and *Tc-arrow*, respectively. In the wild-type blastoderm embryo, posteriorly expressed *Tc-wnt8* [12] showed a limited contribution to the axis elongation process, since only a minor fraction of *Tc-wnt8*^RNAi^ embryos (6%, N = 8) were posteriorly truncated [13]. The frequency of embryos with an axis-elongation phenotype was only enhanced in the double RNAi with *Tc-wntless* dsRNA [13]. On the contrary, the Wnt-coreceptor *Tc-arrow* is an obligatory component for Wnt-dependent developmental processes, including abdominal elongation [14]. For the double knock-down, the same range of phenotypes as in the single *Tc-axin* RNAi experiment has been obtained in both double RNAi combinations. However, the frequency of the DA-phenotype was found to be remarkably higher, 75.5% (N = 62) in *Tc-axin*/*Tc-wnt8*^RNAi^ and 62% (N = 21) in *Tc-axin*/*Tc-arrow* ^RNAi^ embryos, in both of the respective double-RNAi experiments (see for an example Figure 2C; Table 1). Moreover, the expression of marker genes analysed in the combined RNAi experimental embryos showed the same characteristic changes as observed in *Tc-axin* single RNAi embryos: the enhancement of *Tc-caudal*, *Tc-wnt8* and ectopic expression of the hindgut marker *Tc-byn*.

**Figure 4:**
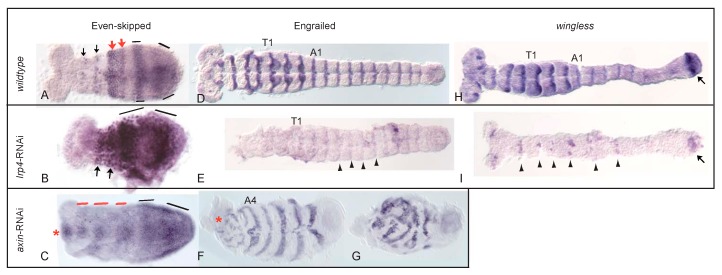
Analysis of segmentation genes in *Tc-lrp4* and *Tc-axin*^RNAi^-embryos. Preparation of fixed embryos. (**A**,**D**,**H**): wild-type embryos; (**B**,**E**,**I**): *Tc-lrp4^RNAi^*-embryos; (**C**,**F**,**G**): *Tc-axin^RNAi^*-embryos. (**A**) Expression of the pair-rule gene Even-skipped (antibody staining). In the wild-type, two broad domains that represent the double-segmented phase are seen in the growth-zone (bars). The split into segmented stripes starts just anterior to the growth-zone (red arrows). The first two segmental eve stripes are about to dissolve (black arrows). (**B**) *Tc-lrp*4-RNAi: Eve expression is stronger when compared to the wild-type and hence, single stripes are hardly visible. (**C**) *Tc-axin*-RNAi: broad domains (black bars) in the growth-zone appear but do not dissolve into segmental stripes; instead, block-shaped domains in the region where the mesoderm forms are visible (red bars). Ectopically, Eve-positive cells are visible in a medial, anterior position (red star). (**D**): Fully segmented wild-type embryo. Engrailed expression demarcates the posterior segmental border. (**E**): *Tc-lrp*4-RNAi: headless embryo lacking clearly identifiable En-stripes but with clearly visible segmental grooves (see arrowhead for examples). (**F**,**G**) *Tc-axin*-RNAi: scrambled En stripes at the anterior part of the embryos (red star in (**F**)). No polarity information is recognizable. (**H**) *Tc-wingless* (*wg*)/*Wnt1* expression (in-situ-hybridization) in the wild-type undergoing axis elongation. (**I**): *Tc-lrp*4-RNAi: remnants of *wg* positive cells are visible in the segmental pattern (arrowheads). Posterior *wg* domain (arrow) seems weaker than in the wild-type but is clearly visible.

To reveal segment formation in embryos with elevated Wnt activity at the anterior, we analysed the expression patterns of the segmentation gene products Even-skipped and Engrailed in *Tc-axin*^RNAi^ embryos. The expression of the pair-rule gene product Tc-Eve in the wild-type embryo developed its segmental pattern from primary, broad, double segmental stripes within the growth zone of the embryo that then split into secondary, segmental stripes (Figure 4A) [28,29]. 

Remarkably, in *Tc-axin*^RNAi^ embryos, Tc-Eve was expressed in young germband embryos along the AP axis in dense blocks in the mesodermal region, ectopically at the anterior and in the growth zone in solid stripes that apparently did not dissolve into secondary, segmental stripes (Figure 4C). The wild-type expression pattern of Tc-En was seen in *Tc-axin*^RNAi^ embryos as segmental stripes (Figure 4F) within the posterior abdomen, but was uncoordinated within the anterior region of the embryo (Figure 4F, star). Stronger affected short embryos display uncoordinated patches of Tc-En expression (Figure 4G).

### 3.3. Structure, Expression and Functional Analysis of Tc-lrp4

The gene TC007146 was identified as the single Lrp4 ortholog in the *Tribolium* genome [14] that codes for a 2042 amino acid long, single-pass transmembrane protein. It is structured as a typical LDL (low density lipoprotein) receptor [30] with 8 LDL-A repeats, followed by 1 EGF repeat, four (1 EGF-5 YMTD) repeats and the transmembrane domain (Figure S2B). Crucially involved in binding WNT proteins are the first two of the YWTD and the EGF domains [31,32,33]. Intracellularly, Lrp4 lacks the highly conserved PPT(S/T)Px(S/T) motifs required for Axin-binding present in the Lrp5/6/Arrow class of WNT-coreceptors [14,34] (Figure S2C). 

**Figure 5:**
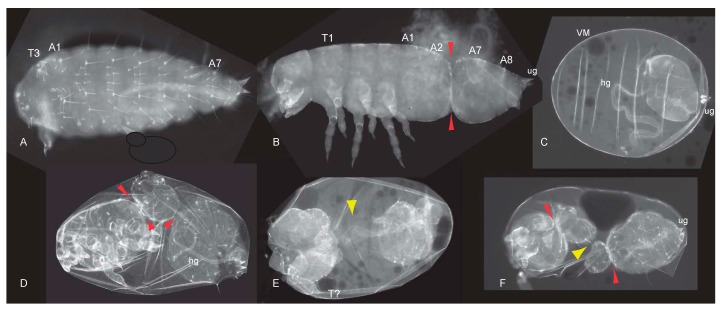
*Tc-lrp4* is essential for patterning the anterior-posterior axis and for segmentation. (**A**–**F**: Cuticle preparations). (**A**) Headless *Tc-lrp4*^RNAi^ larval cuticle. (**B**) *Tc-lrp*^RNAi^ phenotype with a constricted abdomen (arrow heads) missing abdominal segments A3–A6 (“gap-phenotype”). (**C**) Remnant of the posterior cuticle including the urogomphi and the hindgut within the vitelline membrane. (**D**,**E**) Gap-phenotypes are characterized by constrictions and loss of body parts (shown by arrowheads). (**D**) Constrictions between thorax and abdomen and within the abdomen (arrowheads). (**E**) Larval cuticle consists of two pieces (yellow arrowhead indicates absence of connection). The anterior part developed with one pair of malformed legs of unknown identity (T?). (**F**) Cuticle consists of two pieces, each in addition shows constrictions (red arrowheads). Separation is indicated by the yellow arrowhead. A1: abdominal segment 1, HD: hindgut, Lb: labial segment; Mx: maxillar segment, T1: thoracic segment 1. ug: urogomphi. VM: vitelline membrane.

Throughout embryogenesis, and in contrast to *Tc-axin*, *Tc-lrp4* is ubiquitously expressed (Figure S5). To analyse its function during embryogenesis, we performed *Tc-lrp4* knock-down experiments applying maternal RNAi. We observed various segmentation defects along the AP axis but not polarity reversal as seen in *Tc-axin*-RNAi experiments (see above). The variety of *Tc-lrp4-*RNAi cuticle-phenotypes was grouped into three different categories (Figure 5). Class Ia was characterised by missing a single or more gnathal segments up to the loss of the complete head (Figure 5A) and the thorax resulting in an abdomen-only cuticle (Class Ib) (Figure 5C). Class II phenotypes displayed constrictions within the thorax or the abdomen of the larval cuticle. This was accompanied by the loss of a single or a number of adjacent body segments resulting in a gap within the anterior-posterior pattern and, therefore, classified as “gap”-phenotype (Figure 5B,D). The range of gap-phenotypes are displayed in Figure S6. Class III comprised cuticles that consisted of separate pieces with ball-shaped cuticles of unknown segment identity or cuticle remnants with hindgut structures (Figure 5E,F).

### 3.4. Pair-Rule and Segment-Polarity Gene Expression in Tc-lrp4 RNAi Embryos Reveal Instability of the Segment Boundary

In RNAi embryos with impaired Lrp4 function, misexpression of *Tc-caudal* was not as drastic when compared to *Tc-axin* RNAi. Nevertheless, the *Tc-caudal* domain was seen enlarged towards the anterior, covering at most 50% in the growing germband (Figure S7). On the other hand, Tc-Eve expression was seen to be strongly enhanced, and also inter-stripe cells showed to various degrees Eve protein expression so that the stripes appeared to be fused to form a single, large posterior expression domain (Figure 4B). Surprisingly, in *Tc-lrp4* RNAi embryos, the expression of the segment polarity genes *wingless/Wnt1* and *engrailed*, however, are strongly reduced, irregular or absent (Figure 4E,I).

## 4. Discussion

In this work, we analysed in the beetle *Tribolium* the function of Wnt-antagonists that act at the level of the destruction complex and at Wnt-reception. Knocking down the asymmetrically distributed Wnt-inhibitor *Tc-axin* resulted in the polarity reversal phenotype, not observed before. For the first time in an insect, we show that the Wnt-inhibitor Lrp4 acts globally along the anterior–posterior axis.

**The complex expression pattern of *Tc-axin* reveals sites for the modulation of Wnt activity.**

During early embryogenesis, the Wnt-antagonist *Tc-axin* is asymmetrically expressed at the anterior pole of the pre-blastoderm embryo [16]. In addition to this early phase of expression, we show here that *Tc-axin* was also expressed during later embryonic stages in various tissues such as the growth zone of the elongating embryo, in developing appendages and in the abdominal segments. The dynamic expression of *Tc-axin* marks regions and tissues in the embryo where the level of Wnt activity is tightly balanced by the Wnt-destruction complex during embryogenesis. In the pre-blastoderm embryo, *Tc-axin* establishes asymmetry along the AP axis by lowering or eliminating Wnt activity off the anterior pole of the egg. The clearance of Wnt activity by *axin* represents an evolutionary, ancient principle for enabling the development of anterior structures in vertebrates and invertebrates including *Tribolium* [6,16,35]. By regulating the stability of β-catenin, Axin is only indirectly and not instructively required for anterior patterning. One such factor required for anterior development in *Tribolium* and in other invertebrate species outside *Drosophila* [36,37] is exemplified by the *orthodenticle* gene [38,39,40]. 

Later during embryogenesis, *Tc-axin* is expressed in the growth zone of the elongating embryo in a longitudinal stripe. None of the Wnt ligands is expressed in this region (Figure 1C) flanked by *Tc-fgf8* expression [41]. We speculate that, in this way, this part of the growth zone is kept clear of Wnt activity. Interestingly, the expression sites of *Tc-axin* coincide in part with many regions that also express Wnt ligands [12] and hints at the requirement to tightly control the extent of Wnt signaling in tissues like the growth zone and the elongating appendages.

**Embryos with elevated but flattened Wnt activity posteriorise and also develop the double abdomen/double hindgut phenotype.**

**Figure 6:**
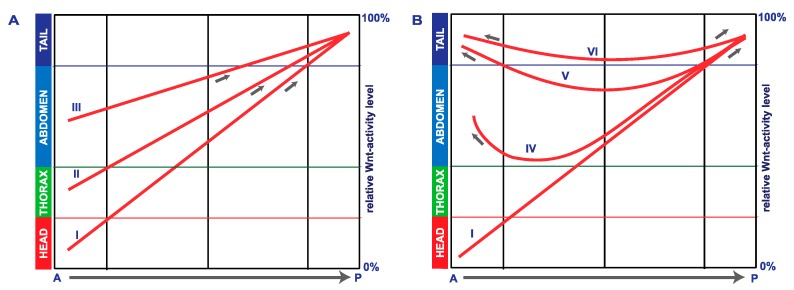
Schematic illustration of Wnt activity along the AP-axis in wild-type- and RNAi-embryos. (**A**) Wnt activity along the anterior-posterior axis is graded from low to high in the wild-type embryo (curve I) resulting in the formation of all pattern elements head, thorax and abdomen. Upon *Tc-axin*-RNAi (weak effect) or LRP4-RNAi, Wnt activity at the anterior becomes de-repressed and thus is elevated in the anterior egg to a level that is not compatible with head- (curve II) nor head/thorax development (curve III). AP-polarity, however, remains normal due to the graded distribution of Wnt activity. (**B**) Embryos that not only miss anterior structures but in addition show development of posterior fate at the anterior pole (curves IV–VI) observed in RNAi experiments knocking down *Tc-axin* alone or in combination with either *Tc-wnt8* or *Tc-arrow*. Such embryos developed polarity reversal of only few abdominal bristles or abdominal segments with reverse polarity at the anterior (curve IV) or a mirror image double abdomen phenotype (curve V). Two opposing hindguts connected by a small cuticle were seen predominantly in the combinatorial RNAi experiments (curve VI), the Wnt-flat^HIGH^ situation.

The loss of anterior larval structures by parental *Tc-axin* RNA interference has been described previously [16] and is shown here (Figure S3B–D). This posteriorisation phenotype is explained by ectopic elevation of Wnt activity in the anterior egg to a level normally found in a more posterior position (Figure 6A, curves II–III) and interferes with anterior pattering. That posterior fate, indeed, has expanded anteriorly, visualised by the ectopic expression of the Wnt target *Tc-caudal* [16] (Figure 3D–F), of *Tc-wnt8* (Figure 3M), and of *Tc-Abdominal B* (Figure 3P). 

In addition, we show here that a substantial proportion of *Tc-axin*^RNAi^ larvae displayed polarity reversal of anterior abdominal segments, the double-abdomen phenotype (Figure 2B–F, Table 1). In embryos with an asymmetric double-abdomen (Figure 2B) the downregulation of *Tc-axin* likely was most effective at the anterior pole, but did not affect the architecture (steepness) of the Wnt-activity gradient in the posterior egg. In such embryos, no recognisable posterior-most structures such as a hindgut or ectopic urogomphi have developed, indicating that Wnt activity at the anterior was too low (Figure 6B, curve V). In mirror-symmetric embryos and larval cuticles, where both abdomens consisted of only 2–3 abdominal segments, the slope of Wnt activity was not steep enough to provide sufficient positional information for abdominal segments of more anterior identity (Figure 2C). In even more extreme cases of the double posterior phenotype, high levels of Wnt activity are even more flattened along the anterior–posterior axis. This “Wnt-flat^HIGH^” situation results in the formation of two posterior centres at both poles that each develops with a hindgut recognised in the cuticle (Figure 2E,F) and the expression of the hindgut-specific marker *Tc-brachyenteron* (Figure 3I–K). The lack of steepness of the Wnt-gradient, and thus the lack of sufficient positional information, only enables the formation of a small cuticle remnant (Figure 2F and Figure 6B, curve VI). 

The rationale of the double RNAi experiments knocking down the Wnt-antagonist *Tc-axin* and at the same time a Wnt-agonist like *Tc-wnt8* or *Tc-arrow,* was to reduce overall Wnt activity and thereby support the flattening of Wnt activity. The fact that slightly more embryos with duplicated posterior structures developed showed that this situation, indeed, destabilises the embryonic axis. The formation of posterior structures and segments could be under the control of the terminal system [42].

Strongly affected *Tc-axin* single-RNAi- as well as in *Tc-axin*/*Tc-wnt8* or in *Tc-axin*/*Tc-arrow* double-RNAi embryos develop with few if any abdominal segments, indicating that high Wnt levels interfere with the segmentation process. We found that the primary pair-rule stripes of Tc-Eve do not resolve into secondary, segmental stripes [28,29] but remain as solid blocks within the embryo along the AP axis (Figure 4C). Obviously, in embryos with **up**regulated Wnt activity, the segmentation process is not primarily affected and repeatedly generates primary Eve-domains. These, however, do not resolve into secondary ones but remain stable and become ectopically placed in the anterior embryo. We have shown, that in *Tc-frizzled*1/2^RNAi^-embryos where Wnt activity is **down**regulated, the transition of pair-rule gene expression from primary to the secondary pair-rule phase is also impaired [14]. Therefore, we conclude, that the level of Wnt activity has to be tightly balanced by agonists and antagonists within the anterior part of the growth zone, and likely in other organs such as the appendages, to ensure normal development. 

Mirror-type duplications of posterior pattern elements in insects by experimental perturbation were explained by the double gradient hypothesis [43,44], but were at that time not understood at the molecular level. We propose that an inhibitor such as *axin* could fulfil the requirement of one component of this system also in other organisms.

**Lrp4 as a Wnt-inhibitor in *Tribolium*?**

In the mouse, LRP4 acts at the neuromuscular junction as a receptor for the motoneuron-derived protein Agrin that is absent from the *Drosophila* genome [45]. An Lrp4 ortholog in *Drosophila* has been identified as a regulator of synaptic physiology in the central nervous system in a Wnt-independent fashion [17]. The reduction of Lrp4 function in *Drosophila* results in the loss of certain synapses but has no implications for the segmentation process [17]. 

Structurally, Tc-Lrp4 could function as a Wnt-receptor that is unable to transmit its signal to the nucleus due to the lack of an Axin-binding domain. In this way, Tc-Lrp4 would function in a dominant-negative fashion to negatively modulate Wnt signaling. In fact, Lrp4 can compete with the Wnt-coreceptors Lrp5/6 for Wnt ligands, as this has been described in the mouse system [46]. The function of vertebrate Lrp4s as a negative modulator of Wnt activity has been also shown for other processes [19,20,21,47]. Also in vertebrates, Lrp4 functions as a receptor for Wnt-inhibitors like WISE, DKK or Sclerotin. It is discussed that Lrp4 bind e.g., WISE, presents it either to the LRP5/6 coreceptor or the Frizzled-receptor, and in these ways competes with Wnt-loaded Lrp5/6 and antagonizes Wnt activity [21]. Since the respective orthologues of WISE, DKK and Sclerotin seem to be missing from the *Tribolium* genome, *Tc*-Lrp4 could function as a dominant-negative WNT co-receptor in *Tribolium*. In this scenario, which has also been discussed for the vertebrate system [21], Lrp4 directly competes with Lrp5/6 for Wnt-ligands and/or Frizzled receptors. Once the Wnt/Lrp4 dimer has formed, less Wnt signal is available and hence, Wnt activity is attenuated. In the experimental situation of downregulating a Wnt-inhibitor, in this case Lrp4, Wnt signaling is expected to become upregulated. That this, indeed, was the case was seen at the anterior through the upregulation of *brachyenteron* and the loss of head- and thoracic structures. At the posterior, the upregulation of Wnt activity results in high levels of *caudal* expression and, as a consequence, elevated *even-skipped* levels were observed in *Tc-axin* (see above) and also, but not as pronounced, in *Tc-lrp4*-RNAi embryos. *caudal/Cdx genes* have been characterized as direct targets of the Wnt pathway [48,49,50] and can be seen as a read-out of Wnt activity. In the *Tribolium* wild-type embryo, the pair-rule circuit that involves *eve*, *runt* and *paired* leads to the activation of *Tc-engrailed* and represses *Tc-wingless,* resulting in an alternating Wg–En expression pattern [51]. Although segments were morphologically visible in *Tc-lrp4*-RNAi embryos, segmental borders are obviously not maintained during development until the cuticle has formed, due to the lack or strong reduction of Wg and En in *Tc-lrp4*-RNAi embryos (Figure 4E,I). Without an established connection between two adjacent segments, neighboured segments might collapse and eventually fuse (“gap-phenotype”), and the cuticle displays severe constrictions when only part of a segment border forms or the larval cuticle falls apart and consists of separate pieces that we observed within the vitelline membrane. 

Since the exact binding partner of *Tc-*Lrp4 has not been identified, other ligands but also other downstream events could be elicited by the activated Lrp4 receptor in *Tribolium* as in *Drosophila* Lrp4 signals through a synapse-specific kinase in a Wnt-independent manner. Interestingly, an Agrin-ortholog has been identified in *Tribolium* [52] that, indeed, could function as a ligand for *Tc*-Lrp4. Taking into account that such a scenario is given for the neuromuscular junction in the mouse, a contribution of Agrin in segmentation in *Tribolium* seems unlikely.

No polarity reversal was observed after *Tc-lrp4*-RNAi. In contrast to *Tc-axin*, *Tc-lrp4* was shown to be ubiquitously expressed throughout the egg and the embryo. In the *lrp4-*RNAi embryo, mRNA became evenly reduced along the complete AP axis to a lower level, so that Wnt activity becomes elevated without altering the steepness of its activity gradient and, importantly, without accumulating at the anterior pole to a level that could result in polarity reversal (Figure 5A; Figure 6A, curve II). The elevation of Wnt activity at the anterior pole above a certain threshold, however, interferes with head and thorax development without altering the overall polarity of the embryo. When the Wnt-activity level rose more in *Tc-lrp4* RNAi embryos, only posterior abdominal segments can form but still exhibited normal polarity (Figure 6A, curve III). A different situation is seen in *Tc-axin*-RNAi embryos where asymmetric expression of *Tc-axin* is crucial for downregulating Wnt activity at the anterior pole of the early wild-type embryo. In the *Tc-axin-*RNAi experiment, anteriorly expressed maternal *axin* is downregulated and results in an increase of Wnt activity at the anterior (Figure 6B, curve IV). Here, polarity information on anterior-forming abdominal segments could, additionally, come from the terminal system [42], resulting in hindgut development at the anterior pole. In the extreme situation, the height and the lack of steepness of the Wnt activity level (Figure 6B, curve V, VI) allows for the formation of only few abdominal segments of posterior-most identity, with reversed polarity also seen in the *Drosophila bicaudal* mutant [53,54] or in *panish*-RNAi *Chironomous* larvae [55] and not for two opposing abdomens with the full set of eight abdominal segments.

## 5. Conclusions

The distinct expression sites of the Wnt-antagonist *Tc-axin* seen in the developing embryo reveal tissues and cells where Wnt signaling possibly needs to become fine-tuned to ensure proper development. Apparently and unexpectedly, negative modulation of Wnt activity likely also occurs close to or coinciding with Wnt-agonist expression sites, such as in a central stripe within the growth zone or in the ventral appendage. Experimentally, we found that downregulation via maternal RNAi, *Tc-axin* not only led to the repression of anterior development but, in addition, to the formation of an ectopic tail at the anterior pole with reversed polarity and a hindgut, resembling the *Drosophila bicoid* or *bicaudal* mutant phenotype. For the first time, we could show that an insect Lrp4 orthologue functions along the anterior–posterior axis, coordinating axis formation and segmentation possibly by antagonising Wnt signaling. This work broadens our view on the modulation of Wnt activity by the negative Wnt-regulators Axin and LRP4 to ensure proper embryonic development.

## Figures and Tables

**Table 1:**
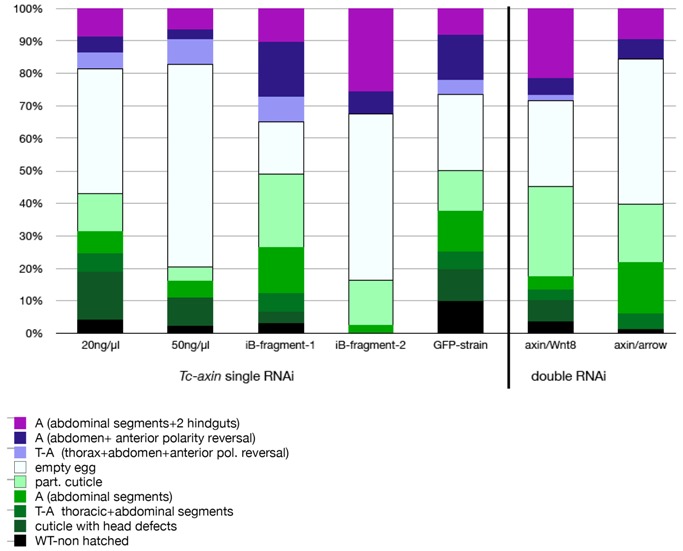
Quantitative analysis of *Tc-axin*-RNAi.

Numbers (N =)	*axin*^RNAi^_20	*axin*^RNAi^_50	iB-04108-1	iB-04108-2	*axin*^RNAi^ GFP-Strain	*axin*/*Wnt8* Double RNAi	*axin*/*Arrow* Double RNAi
**WT**	93	91	44	-	167	126	25
**K-T-A**	15	8	4	-	7	19	1
**T-A (−)**	6	-	6	-	4	9	10
**A (−)**	7	5	15	1	9	12	34
**part. cuticle**	12	4	24	6	9	80	39
**T-A (+)**	5	7	8	-	3	5	-
**A (+)**	5	3	18	3	10	15	12
**A (+2 hg)**	9	6	11	11	6	62	21
**cuticles with polarity reversal**	19	16	37	14	19	82	33
**affected cuticles**	59	33	86	21	48	202	117
**all cuticles**	152	124	130	21	215	328	142
**empty eggs**	39	58	17	22	17	76	97

Green indicates cuticles missing anterior structures; pink highlights the cuticles showing polarity reversal; (−) cuticles without polarity reversal; (+) cuticles displaying polarity reversal.

## Supplementary Materials

The Supplementary Materials are available online at www.mdpi.com/2221-3759/5/4/10/s1.
