# Supplementary material for: The Roles of the Wnt-Antagonists Axin and Lrp4 during Embryogenesis of the Red Flour Beetle Tribolium castaneum
Source: J Dev Biol. 2017 Oct 15;5(4):10. doi: 10.3390/jdb5040010 (PMC5831798; doi:10.3390/jdb5040010)
Supplement: Supplementary file 1 [file jdb-05-00010-s001.pdf]

Supplementary Materials: The Roles of the Wnt-Antagonists Axin and Lrp4 during Embryogenesis of the Red Flour Beetle *Tribolium castaneum*

Romy Prühs, Anke Beermann and Reinhard Schröder

A *Tc-axin*
TC006314

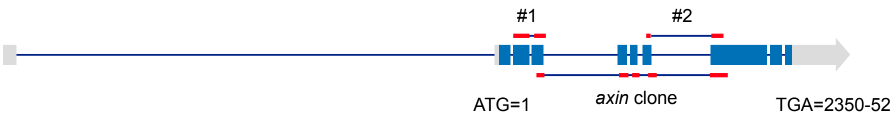

B iB_04108_1
position 184-695

```
TTTGATTTCGACGATGGTAGCAGTTACAGGCCTACTTCGCGGCCTTCGTCGCGCCACGGGAAACCGCCGGCCTACCTC
GCATGGGCCGAATCGCTGCACAATCTGCTGCAGGATTCGGACGGTGTGAAATTATTCTTGCGTTACCTCGAATCGGAA
GGGCCCCATCATGCCGACACGTTGCGCTTTTGGTTCGCTTGCGAAGGCTTGAAGAACCACGATCAAGTCGAAAATATA
CAAAAGTTGGTTAAATTGATCTACAAAACGTATTTTTTGAAATCGGCCCTACAAATCAACGACGATCTGAAGAAACAA
ATAATGAGAAATTTAAAATCGCCCCAGTGCCTTGAACCACCCATTACTCTGTTCGACGAGGCCCAAGCACAAATCGAA
AACCTTATCAATAAAACTACTTATCCCAACTTCCTCAAAAGCGACATCTATCTGCAATATGTCCAATCGGTCGAGAAT
GCGAGCAGCGACGACTTCAACAGCGAATCGAGCTCTTCCAGCAG  (512bp)
```

C iB_04108_2
position 990-1213

```
GAGGCAAGATAGCGAAATGCAGTCGCTTAGTAGTCATTCAGATGCGAGGACCGAATCGGACAATATGTCCTTAAATGA
GAGCAATGTTGACGGCAGACCAGGTCGTTCTTTACGCAAATCCATCGAAGAGAAACAAATGCGTCAATTCGCCCGTAA
TAAAGAAGTAAACATGAACCAACGTTTCATCCCACGAACCCAACGCATGGATGTGGGTAACAATTGCC (224bp)
```

D *axin*-clone
position: 523-1344

```
TGCCTTGAACCACCCATTACTCTGTTCGACGAGGCCCAAGCACAAATCGAAAACCTTATCAATAAAACTACTTATCCC
AACTTCCTCAAAAGCGACATCTATCTGCAATATGTCCAATCGGTCGAGAATGCGAGCAGCGACGACTTCAACAGCGAA
TCGAGCTCTTCCAGCAGTTCCCTCAGTAACAAAGACTTGGGGAACTTGGCCAGCGGTTTGGGCCCCCTTCCCACGCTC
CACGAAGGCATGGAATTCAGTCTCAACACCCAGCAGCCCGTCATCCTAACGCCAGGTGCAGCCAGTGTCAGTACGGGT
TATCACACGCCAACAGTTCGCCTCACCAAAGACATGCTCCTCCGATCTCAAAAGCATCGCGCTCTCGACATCCGCCCC
AAATCCGAGACCTATGCTGGTTTGATATACCGAAGCAGTGGGGCTCACGTCGCATACAATTCCTACAACCCGGTCTCG
AGGCAAGATAGCGAAATGCAGTCGCTTAGTAGTCATTCAGATGCGAGGACCGAATCGGACAATATGTCCTTAAATGAG
AGCAATGTTGACGGCAGACCAGGTCGTTCTTTACGCAAATCCATCGAAGAGAAACAAATGCGTCAATTCGCCCGTAAT
AAAGAAGTAAACATGAACCAACGTTTCATCCCACGAACCCAACGCATGGATGTGGGTAACAATTGCCTGCCAATCGAA
GAACACGCCGCTCTCTTGATAAAAAAGCTCGAAAATCTCAAACGCGAACAGGAGAACCAGGAGTTGTTCAAGCGCCGA
TTGAAAGAAGGCGATTCGCTGAGTTCGCTGGATGACAAATTC (822bp)
```

Figure S1. Structure of *Tc-axin* gene. The position (**A**) and sequences (**B**–**D**) of the non-overlapping iBeetle constructs #1 and #2 and the clone used for the generation of dsRNA.

A *Tc-lrp4*
TC007146

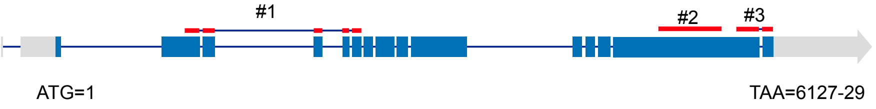

B Tc-LRP4

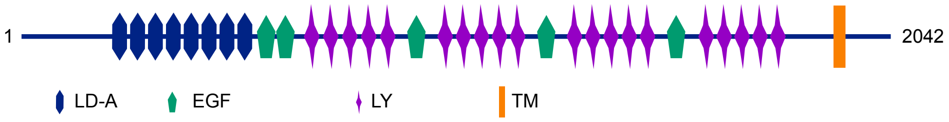

C

```
MKRDFDKPYGDVVKFCLLLGCLVSCVRCAGEATSNPLITAPPHRGAARQMYGSGSLRPPGTKPIYATGQRQHGVRTTISPDQINPRRIFLPPLQ
SYPIVPYPIPMREYDRYSGYPIFNYKDFLSSLPSSSNMDRPSNRGMYGGRLEYSEGRPNHGLVRTRYPFDYGFIPGRGLPPDPSPRRLGRFPSK
SGFLNKNNDMKLKFPEEDDEKQLEETYGHPCGEGCAPGQFLCVASCICINEKNRCDGQEDCDNEEDELECGEIESQRNAKCEANENFIRCPGSG
KCILRSWLCDGDDDCGDFSDETHCGYTVNCSSDQFECANGLCIPKTWLCDNDNDCKDFSDELNCTKTAGCSEDEFECSDESCISLSWKCDRQVD
CSDGSDERDCDITPPYCNEGEFECASKQCIKLKFKCDGDKDCDDWSDEDDCGKAPGNCVSGEFKCNSGKCIPERYRCDKQQDCEEKEDEANCDY
SLARSCSPDEHTCSNGACILKTWVCDGYNDCPQGEDESKCEIVCDESKFSCSGHGINDTATEFCISRKHVCDGKKDCPKGEDESGCPTKRECEK
HTNCTQLCITTVDGRKGCDCIPGYKIASDGITCEDINECLYATDPVCSQTCNNTQGSFRCGCMTGYVLRPDLRTCKAIGDPPTLLFANRIDIRQ
VSLSNAKYTPILKGLHNAIALDYHYDKKLIFWSDVSMDVIRRATMNGTEITDIIKWGLQSPGGVALDWIHDLIFWTDSGTRRIEVASLDGKHRA
IIAGNDIDKPRAITVHPGEAYVFWTDWGPNPKIERAEMDGSNRRSIINESVFWPNGLTLDYTSNRIYWADAKHNVIETALFNGNDRRKVISKGL
PHPFALTIFEDAIYWTDWHTKSISTANKATGAGLRTIHSHLHFPMDIHSYHPQRQPKYSNRCGGNNGGCAHLCLPNRKSYTCVCRMGQKLKSDK
KSCQKPEKLLIFARKKDLRIKHLDNNAVHQHSLVIPLDGVKSAVAIAWDSESDYIFWTDVERDTINRALWNGSNQEVIVYSNIVTPAGLALDWL
TNKIYWTDADTTRIEVAKTNGEMRSLLVWDNLYKPRDIVVDPIGGYMYWSDWAETPKIERAAMDGTMRSVIVSSNLTWPNGLAIDHSAGKIYWA
DGGTKAIECSNFDGSKRKTIIEGPELPHPFGLDVFENYIYWTDWRTLTIERANKNTGLNRTVLASDITDLMDVRVFHRNRKMIKTPCGTKNGGC
THLCLLKPKNNHTCACPTGIKLEEDGKTCANGPINFLILAHRNVIRQISLDVPYIADVVLPLPPLKLVTSVDVDRKTGEIYWIDQAEDVIQKST
RDGQNSKLIIMHKLQTPEGIAVDSTGRKIYWTDGERNSVEVAELDGSNRKVLFSTNLDNPRAITLHYHHGLMFWSDWGKKAKIEVAQMDGTKRK
ALITDGLVWPNGLAIDRPTERLYWNDAKLHTIESSDLKGHDRKIIVTEAPHPYGLVVVGNHIYWTDWQTQALHRADKTNGTDKAKIREKLDGLM
DVRSVQSDNIAENACGGNNGGCSHLCLRNSGGFSCACPTGIKMSKANPKVCEKQPSTYLLLATRFALNRISLDTDDLWDVTLPIEDVNNVIDVD
FHWEKRLIYYTDIGNNVIQSVNMFNLSDTRNIVHSNLSSPDGIAVDWVANNIYWTNTGNKVIEVAKIDGSNRKTIVSTNLQDPRSIAVFPRKGY
LYWTDWGKPKIERSFLDGSGRRILINEQLSFPIGLAIDFVDKRLYWIDATLNEERIETSDLHGRNRVVLDIPQTHPFSLTQFGEHIYWTDWVHK
TVARADKTSGKDVVIVRSYLEAAMGVTMVTESRQTGWNPCAANNGGCPYLCFFKQKNYTCGCPNDHVKCVEGEPKNPVPNKCPSGSYKCDYDDD
DDEDLYNPSNVRDLDETPEQSSGEHWNRFYIMTLVPMLCIILLCIILVAFLLFKKGKKKYLYATGRSFSNPNYYSSGNEPGVAAASGDRKQFIW
KKLKYDKSQERVYEETAGTNSPEIASLIPTILTPCSSNCETVTPELERSPSVTPLHKIEMVQALQADA
```

Figure S2. Structure of the *Tc-Lrp4* gene and protein. (**A**) Exon–Intron structure and position of the non-overlapping PCR fragments used for RNAi: 16 Exons, red-bars show the position of the PCR-

fragments. (**B**) Structure of the protein domains predicted with SMART (EMBL). LDA: low-density lipoprotein receptor domain class A; EGF: epidermal growth factor-like domain; LY low-density lipoprotein-receptor YWTD domain; TM: transmembrane domain. (**C**) Predicted protein sequence highlighting the YMTD repeats.

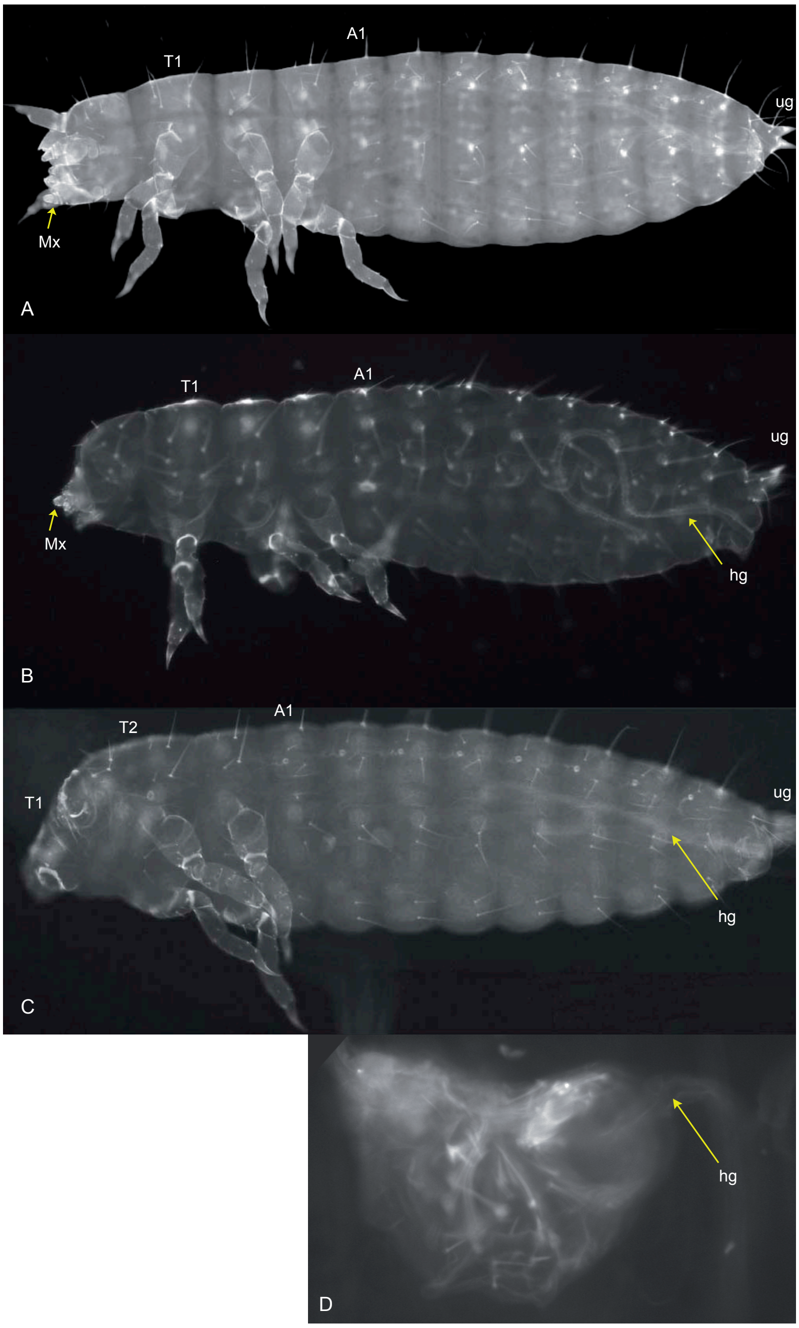

Figure S3. Loss of anterior structures in *Tc-axin*^{RNAi} embryos—weak phenotypes. Cuticle preparation, autofluorescence viewed with ultraviolet (UV) illumination. (**A**) Wild-type cuticle (as in Figure 2A). (**B**) Head structures anterior to the maxilla (mx) are missing. (**C**) Head completely absent; appendages of thoracic segment 1 shortened. (**D**) Strongly reduced abdomen consisting of few segments with the hindgut (hg) sticking out Mx: maxilla; T1: thoracic segment1; A1: abdominal segment 1; ug: urogomphi; hg: hindgut; anterior points to the left, dorsal is up.

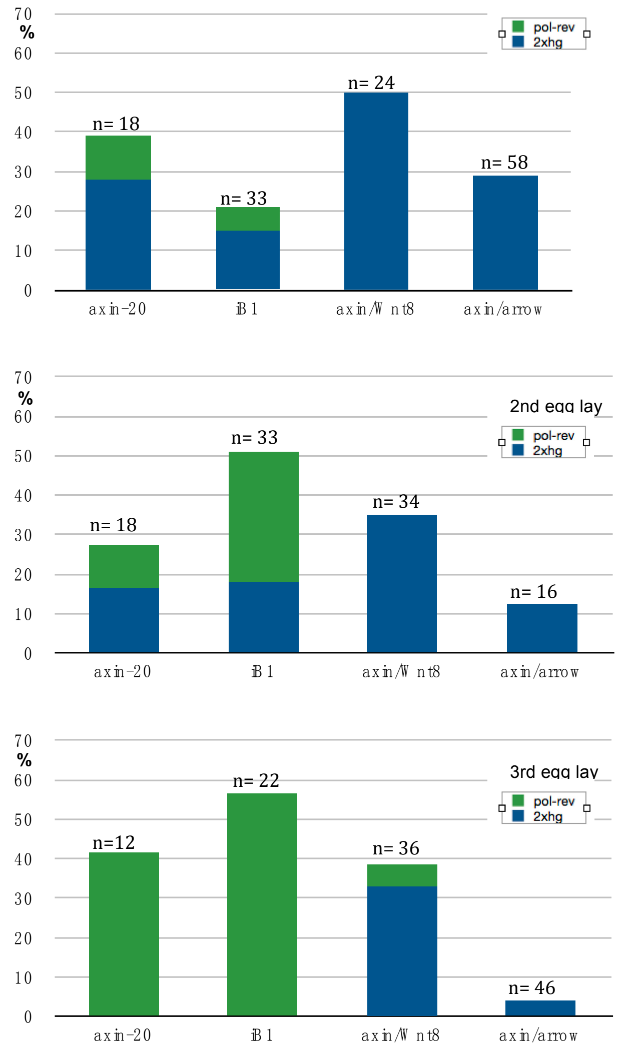

axin20: 20ng/µl; axin dsRNA; **iB1**: dsRNA-fragment (Eupheria) 50ng/µl; **axin/Wnt8**: dsRNA concentration: axin: 20ng/µl axin ; Wnt8: 600ng/µl; **axin/arrow**: 20ng/µl axin ; Wnt8: 100ng/µl

Figure S4. Larval cuticles showing anterior polarity reversal. Quantitative representation of cuticles showed a mild or more severe double abdomen phenotype or "anterior reversal" (green bars), and the development of a second anteriorly positioned hindgut (blue bars). Discrimination was done in relation to the number of egg-lays, concentration of dsRNA applied, the fragment used for dsRNA production and single or double RNAi experiments.

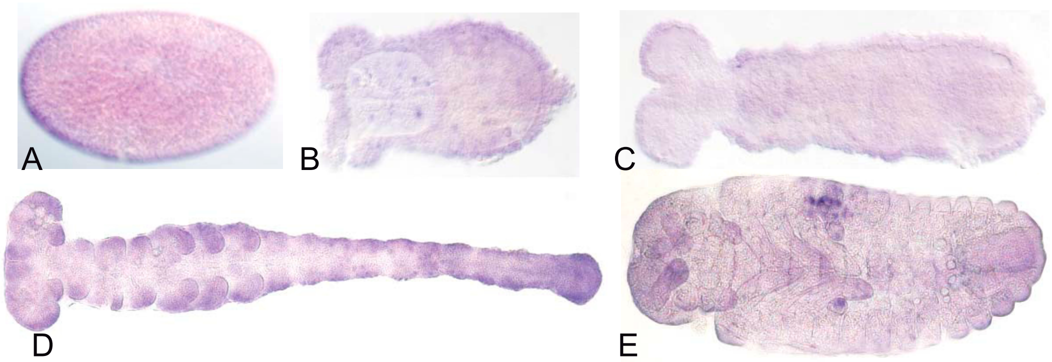

Figure S5. *Tc-lrp4* is ubiquitously expressed during embryogenesis. (**A**) Blastoderm egg, (**B**) Germ anlage, some dots likely representing yolk particles are stained, (**C**) elongating germband, (**D**) nearly fully elongated embryo, (**E**) Retracted germband pseudopodia and yolk particles show unspecific staining.

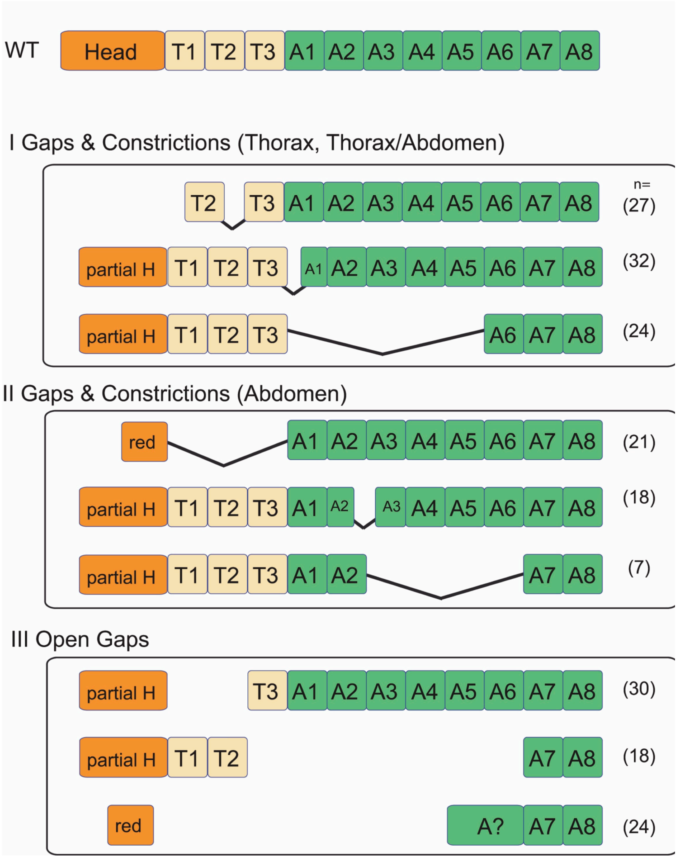

Figure S6. Gap-phenotype in *lrp4*-RNAi cuticles. Schematic representation of the cuticles showing constrictions and/or gaps in the thorax or between thorax and abdomen (Class I), in the abdomen (Class II) or consisting of two pieces (Class III). Number (n =) of observed cases are given in parentheses; red: head strongly reduced to a ball-shaped sphere.

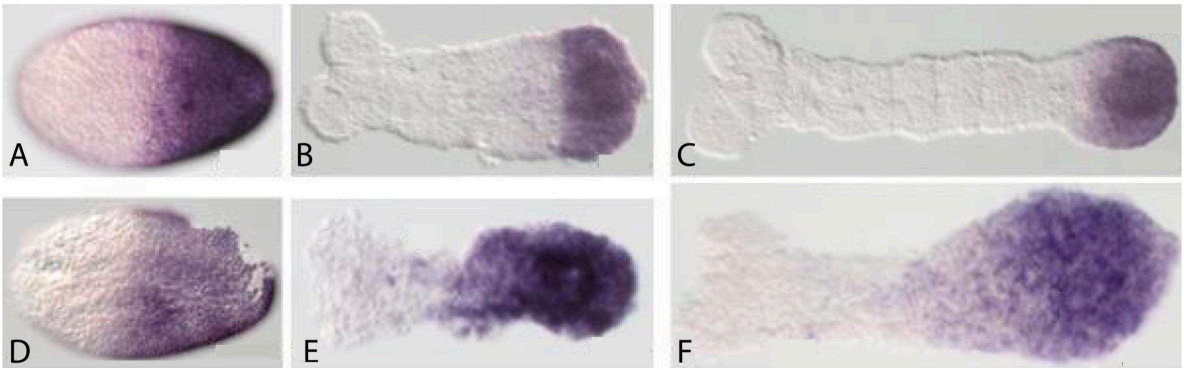

Figure S7. Upregulation of the posterior marker gene *caudal Tc-lrp4* ^{RNAi} embryos. In blastoderm-stage *Tc-lrp4*^{RNAi} embryos, the *Tc-cad* domain was found to be slightly expanded towards the anterior (**D**) when compared to the same stage in the wild-type (**A**). During germband extension, *cad* expression that in the wild-type was strictly confined to a region within the growth-zone (**B**,**C**) was found enlarged up to about 50% embryonic length in *Tc-lrp4*^{RNAi} embryos (**E**,**F**).

Tc-lrp4 RNAi injection series

Table S1. *Tc-lrp4*-dsRNA-injections (without 800 ng, s. Table S2).

Conc. dsRNA	340 ng/µL	1500 ng/µL	1600 ng/µL *	2036 ng/µL	2500 ng/µL	3000 ng/µL
wildtype	2% (6)	0.7% (4)	2.8% (13)	8% (24)	0.7% (1)	2% (18)
empty egg	78% (201)	75.5% (428)	77.8% (362)	69.2% (207)	88.1% (126)	63.6% (548)
phenotype	20% (52)	23.8% (135)	19.4% (90)	22.8% (68)	11.2% (16)	34.4% (296)

* Injection with RNAi-Template #2, s. Figure S1A.

Table S2. *Tc-lrp4*-dsRNA-injections (800 ng/µL), 6 independent experiments.

	800 ng/µL	800 ng/µL 2*	800 ng/µL	800 ng/µL	800 ng/µL	800 ng/µL
wildtype	2.8% (2)	4.6% (18)	1.4% (8)	1.5% (10)	0.4% (3)	5.1% (14)
empty egg	61.9% (44)	70.8% (276)	69.4% (391)	82.1% (550)	83% (568)	58.8% (161)
phenotype	35.3% (25)	24.6% (96)	29.1% (164)	16.4% (110)	16.6% (115)	36.1% (99)

2*: iB-fragment (= fragment #3) synthesised by Eupheria.

Table S3. *Tc-lrp4*-dsRNA-injections: Classification of observed phenotypes.

	340 ng/µL	800 ng/µL	1500 ng/µL	1600 ng/µL	2036 ng/µL	2500 ng/µL	3000 ng/µL	Σ
I	3.8% (2)	11.7% (71)	6% (8)	13.3% (12)	25% (17)	6.3% (1)	22% (65)	13.5% (191)
Ia	23.1% (12)	21% (128)	20.7% (28)	22.2% (20)	36.8% (25)	18.7% (3)	26% (77)	23.4% (331)
II	9.6% (5)	15.6% (95)	23.6% (32)	12.2% (11)	5.9% (4)	0	18.6% (55)	15.8% (223)
III	63.5% (33)	51.7% (315)	49.7% (67)	52.3% (47)	32.4% (22)	75% (12)	33.4% (99)	47.3% (669)

Class Ia: head defects only; Class Ib: missing head + part of the thorax or head + complete thorax; Class II showing constriction, deletion of segments or completely separated pieces of cuticles; Class III: unstructured pieces of cuticles.
